# Data of simulation model for photovoltaic system's maximum power point tracking using sequential Monte Carlo algorithm

**DOI:** 10.1016/j.dib.2023.109853

**Published:** 2023-11-26

**Authors:** Alhaj-Saleh A. Odat, Moayyad Shawaqfah, Fares Al-Momani, Bashar Shboul

**Affiliations:** a Department of Renewable Energy Engineering, Al Al-Bayt University, Mafraq, Jordan; b Department of Civil Engineering, Al Al-Bayt University, Mafraq, Jordan; c Department of Chemical Engineering, college of Engineering, Qatar University, Doha, Qatar

**Keywords:** PV simulink replication model, Simulation of sequential Monte Carlo, Comparison of maximum power point tracking techniques, Dynamic partial shading weather conditions, Random irradiance and temperature waveforms for PV systems

## Abstract

This article outlines the input data and partial shading conditions employed in the replication model of Sequential Monte Carlo (SMC)-based tracking techniques for photovoltaic (PV) systems. The model aims to compare the performance of classical perturb and observe (P&O) algorithm, particle swarm optimization (PSO) algorithm, flower pollination algorithm (FPA), and SMC-based tracking techniques. The mathematical design and methodology of the complete PV system were detailed in our prior research, titled "Dynamic and Adaptive Maximum Power Point Tracking Using Sequential Monte Carlo Algorithm for Photovoltaic System" by Odat et al. (2023) [1]. The provided data facilitate precise replication of the output, saving significant simulation time. Additionally, these data can be readily applied to compare algorithmic results referenced by (Babu, T.S. et al., 2015; PrasanthRam, J. et al., 2017) [2,3], and contribute to the development of new processes for practical applications.

Specifications Table

**Table:** 

Subject	Energy
Specific subject area	Solar energy
Data format	Raw, Dynamic, STC, Filtered, Analyzed
Type of data	Table, Image, Chart, Graph, Figure
Data collection	The Data have been acquired from the Refs. [2,3]. In addition, some data were generated using Matlab/Simulink considering actual environmental parameters. Moreover, the simulation was conducted by the tool of Matlab/Simulink (2019). The experimental results was carried out by Boost converter designed by Ref. [4].
Data source location	Department of Renewable Energy Engineering, Al Al-Bayt University, Mafraq, Jordan.
Data accessibility	https://data.mendeley.com/datasets/h5ssnmxfxw/1
Related research article	Odat, AS., Alzoubi, O., Shboul, B. et al. Dynamic and Adaptive Maximum Power Point Tracking Using Sequential Monte Carlo Algorithm for Photovoltaic System. Arab J Sci Eng (2023). https://doi.org/10.1007/s13369-023-08023-0

## 1 Value of the Data

• This dataset facilitates direct comparisons with alternative algorithms employed for tracking the maximum power point within photovoltaic (PV) systems, streamlining the process of evaluating performance across diverse weather conditions.
• These dataset findings serve as valuable inputs for implementing and conducting real-world assessments of maximum power point tracking (MPPT) controllers known for their heightened efficiency.
• The acquired dataset holds utility in training MPPT controllers via diverse intelligent control methodologies such as Sequential Monte Carlo techniques.
• The dataset's significance lies in its capacity to enable a comprehensive analysis of PV system behaviour when subjected to abrupt fluctuations in solar irradiance and operational temperature of the PV module.

## 2 Data Description

This article provides numerical data, figures, tables, actual irradiance readings data, and Matlab/Simulink design functions regarding a photovoltaic (PV) system employing a Boost converter and operating under rapid and dynamic weather conditions (i.e. solar irradiance, temperature, and partial shading). The simulation was conducted using Matlab/Simulink on a computer equipped with a Core i7 processor and the Win10 operating system. The methodology of the algorithm consisted of using recursive Bayesian, specifically, sequential Monte Carlo technique (SMC) to predict the next best duty cycle for the Boost converter to generate the maximum power point for each instance of time [5]. Furthermore, the system's effectiveness was confirmed through validation both under standard test conditions (STC) and varying weather conditions of irradiance, temperature, and partial shading (simple, moderate, severe, and dynamic shadings). The results demonstrated a remarkable performance and reliability with the proposed approach [1].

• Solar Radiation, Temperature, and Partial Shadings Profiles used to evaluate the SMC-MPPT Algorithms

The following profiles provide an insight on the severity of evaluation condition that the SMC-MPPT algorithm went through to ensure its capability in predicting the maximum power point (MPP) at all times. These profiles are crucial to evaluate the algorithm, and they range from simple STC with no partial shading on the PV farm to sudden and abrupt changes in solar radiation and temperature profiles while having dynamic and random partial shading weather conditions.

• Input Data Assessment Profiles

**Fig. 1:**
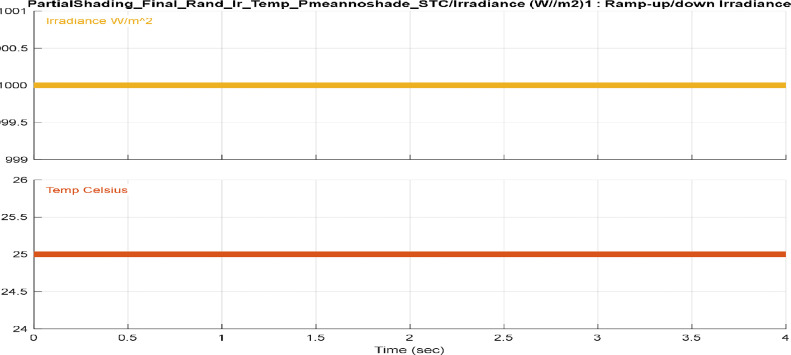
Solar radiation and temperature inputs at STC.

**Fig. 2:**
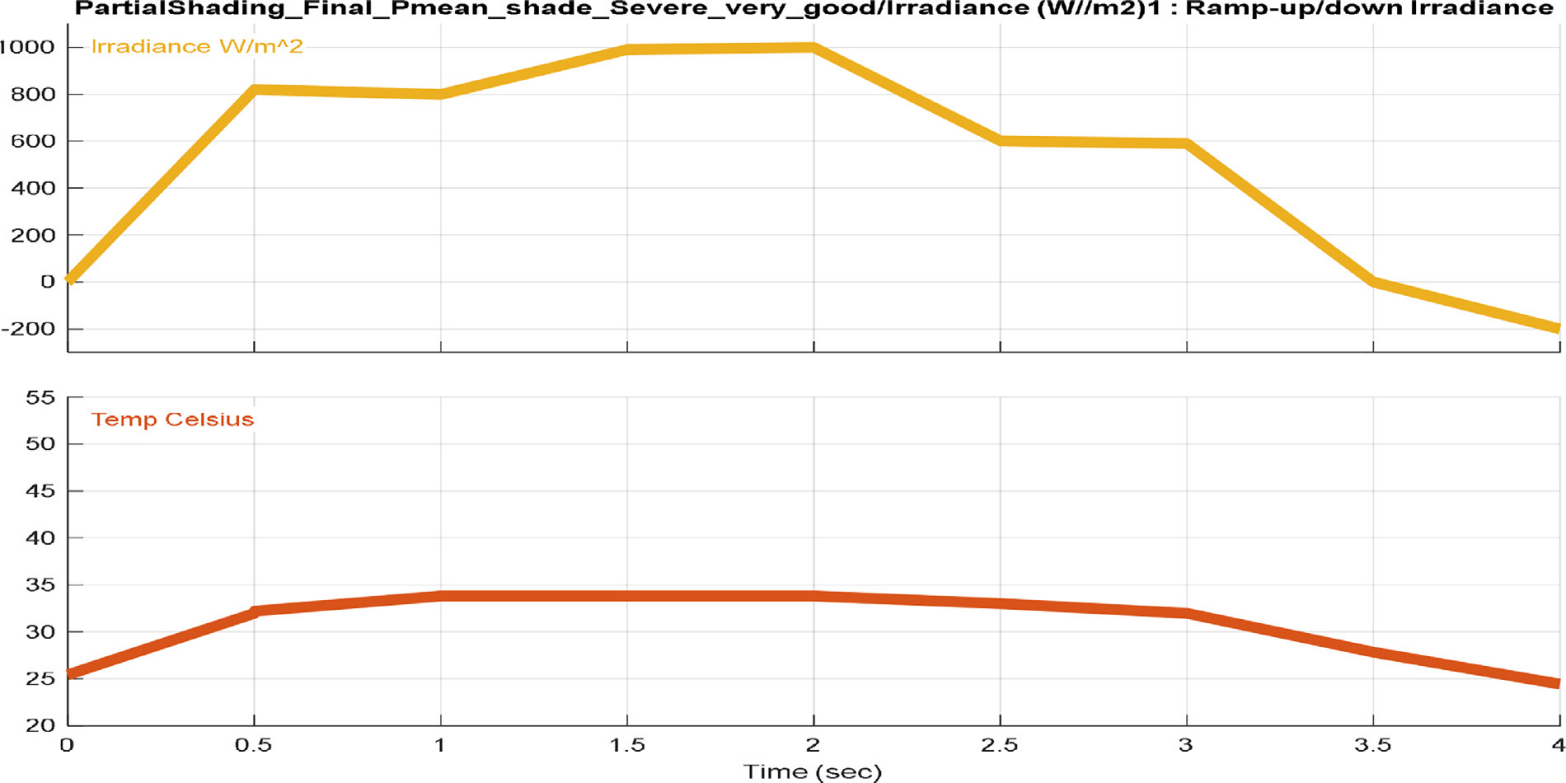
Ram up/down solar radiation and temperature inputs.

**Fig. 3:**
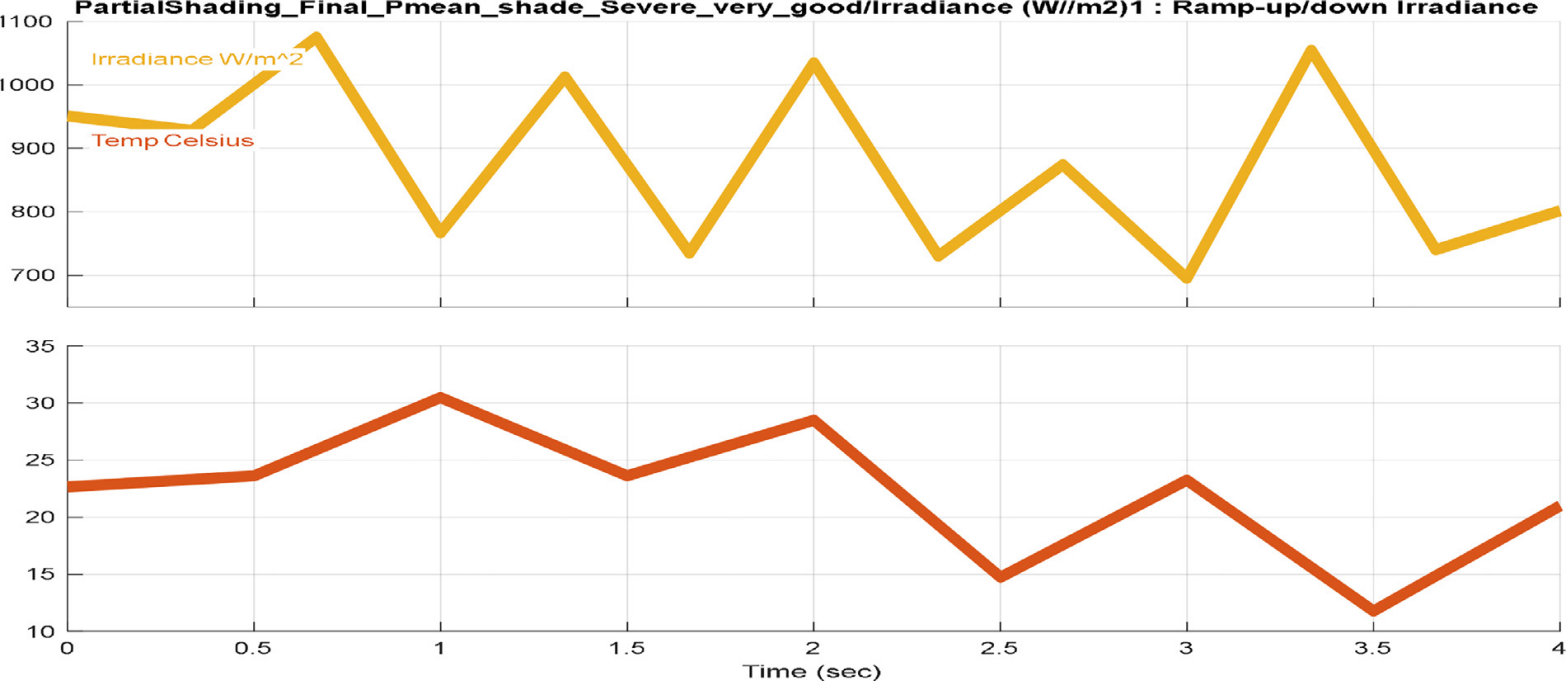
Sudden and abrupt solar radiation and temperature inputs variations.

**Fig. 4:**
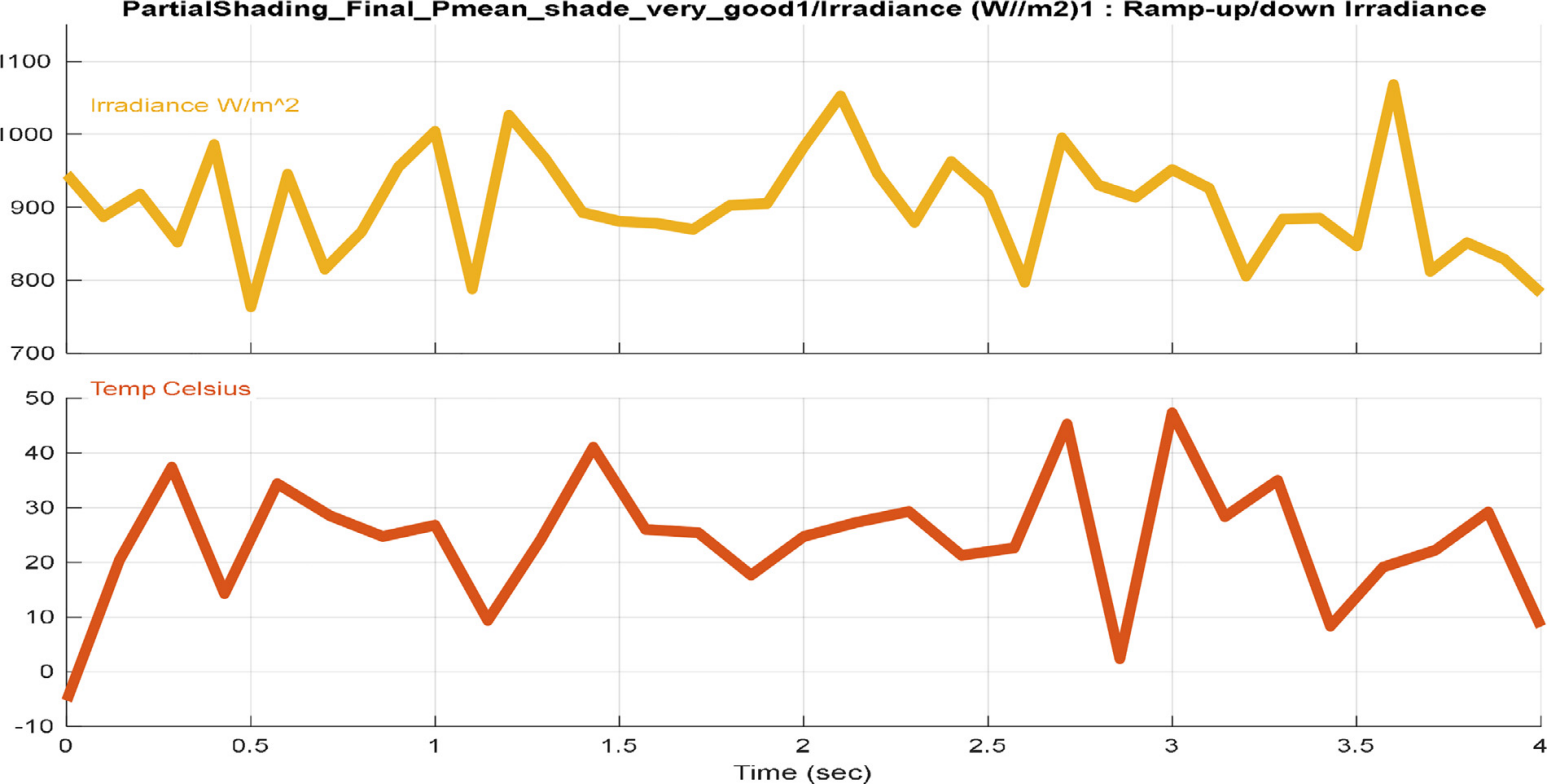
Extreme solar radiation & temperature variation inputs.

Fig. 1, Fig. 2, Fig. 3, Fig. 4 provide different profiles scenarios considered in the assessment for the solar radiation and temperatures input profiles.

1. Solar Radiation and Temperature at Standard Test Conditions (STC)

Fig. 1 illustrates the standard test condition (STC) input profile scenario used as input for the Photovoltaic system reference in [1]. In this figure, the input irradiance remains constant at 1000 W/m², and the temperature is maintained at a constant 25 °C, with no partial shading affecting the system. STC provides a standardized benchmark against which the performance of PV systems can be evaluated consistently. This baseline allows for comparisons between different systems, configurations, or technologies, offering a reliable measure of efficiency. In addition, Testing under STC ensures that the PV system is calibrated and verified under ideal conditions. This step is crucial for establishing the system's baseline performance and validating its adherence to expected standards.

2. Simple Ram up/down Solar Radiation and Temperature

Fig. 2 illustrates a solar radiation and temperature waveforms with a simple variation (ramp-up/ramp-down), depicting the movement of the sun from dawn to sunset on a clear, sunny day. This scenario is presented without any partial shading affecting the PV system. The waveforms capture the natural progression of solar radiation and temperature throughout the day, accounting for the internal temperature rise of the solar panels.

3. Sudden Varying Solar Radiation and Varying Temperature.

Similar to Figs. 2 and 3 represents solar radiation and temperature waveforms with a sudden variation in both. This profile scenario is designed to test the MPPT-SMC algorithm's capability to detect sudden, unexpected changes in weather, such as a rapid drop in temperature due to wind chills, unexpected storms, or cloud movement.

4. Extreme Solar Radiation & Temperature Variation Inputs.

The algorithm's capacity was evaluated in the ultimate scenario, involving random variations in solar radiation and temperature affecting the PV system (see Fig. 4). This case pushes the algorithm to its limits, assessing its ability to navigate through severe changes without encountering unexpected loops or getting stuck in unforeseen states.

5. Experimental Solar Radiation Inputs.

Experimental solar radiation measured data are provided in excel formats in Ref. [6].

• Array I-V & P-V Characteristics

Understanding the Current-Voltage (I-V) and Power-Voltage (P-V) relationship is very crucial to understand the effect of solar radiation and temperature on any PV system. This section provides an overview of how the maximum power point (MPP) moves on the graph when the solar radiation and PV array's temperature increase or decrease. Figures [[5], [6]] provide an overview explanation of the effect of varying solar radiations and temperature values on each PV array system.

1. Array I-V and PV Characteristics while Varying Solar Radiation and Fixing the Temperature at (25 °C).

**Fig. 5:**
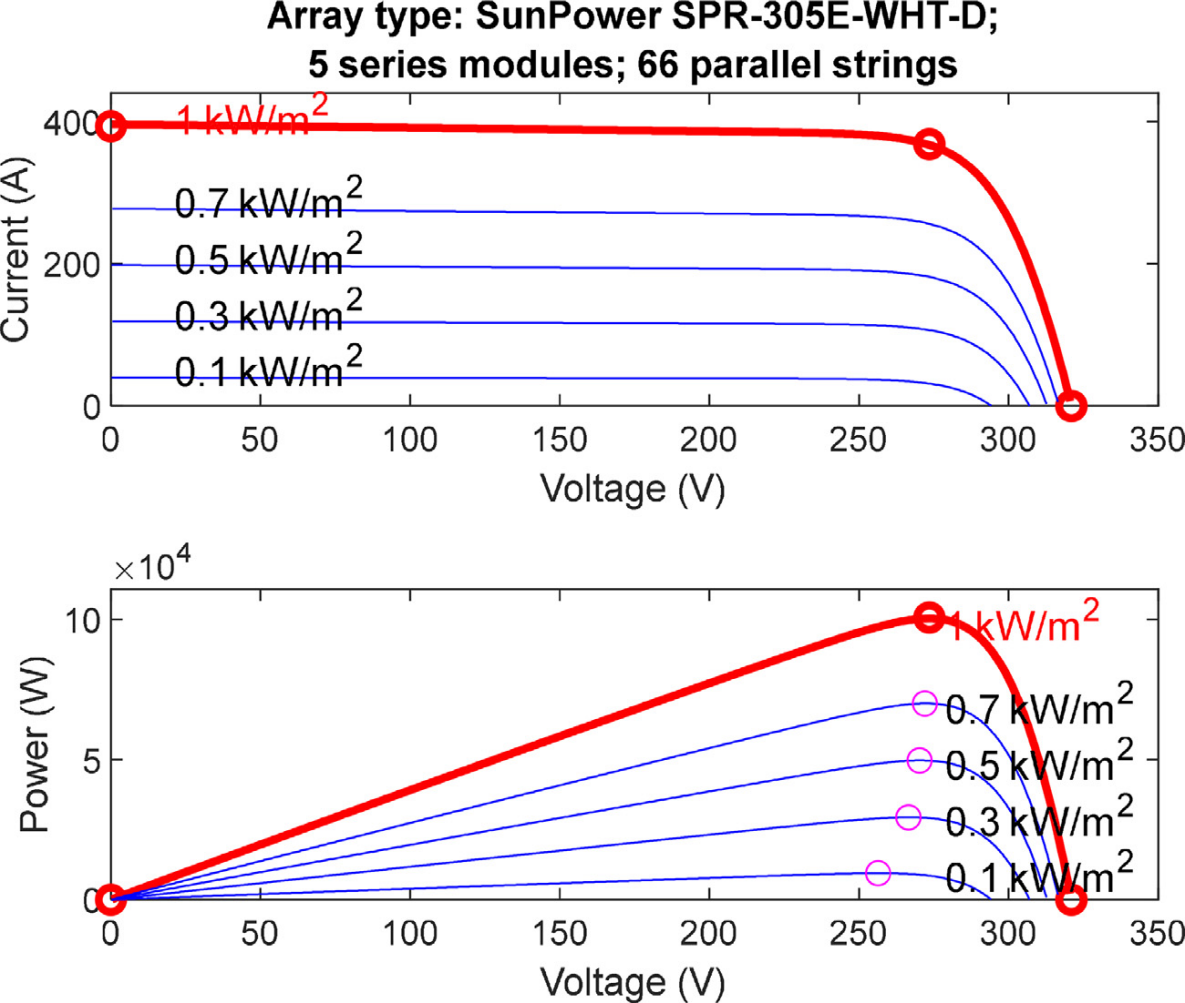
I-V & P-V characteristics (varying solar radiation at temperature (25 °C)).

Fig. 5 explains the effect of changing the solar radiation values of the system, while maintaining a constant PV array temperature of (25 °C). Varying solar radiation while maintaining a constant temperature has a significant impact on the electrical characteristics of photovoltaic (PV) cells, as depicted in the I-V (current-voltage) and P-V (power-voltage) curves. Solar radiation directly influences the amount of sunlight reaching the PV cell, affecting the number of photons available for the generation of electron-hole pairs. As solar radiation increases, the I-V curve exhibits a proportional rise in current, reflecting heightened electron flow. Similarly, the P-V curve demonstrates an increase in power output, reaching its peak at the Maximum Power Point (MPP). Conversely, reduced solar radiation leads to decreased current and power output. Understanding these variations is crucial for optimizing the performance of PV systems under changing environmental conditions. It enables engineers and researchers to design and implement strategies that enhance energy capture efficiency, making PV cells more adaptable to different solar exposure levels while maintaining a constant operating temperature.

2. Array I-V and PV Characteristics while Varying the Temperature and Fixing Solar Radiation at ($1000\;\;W/m^{2}$).

**Fig. 6:**
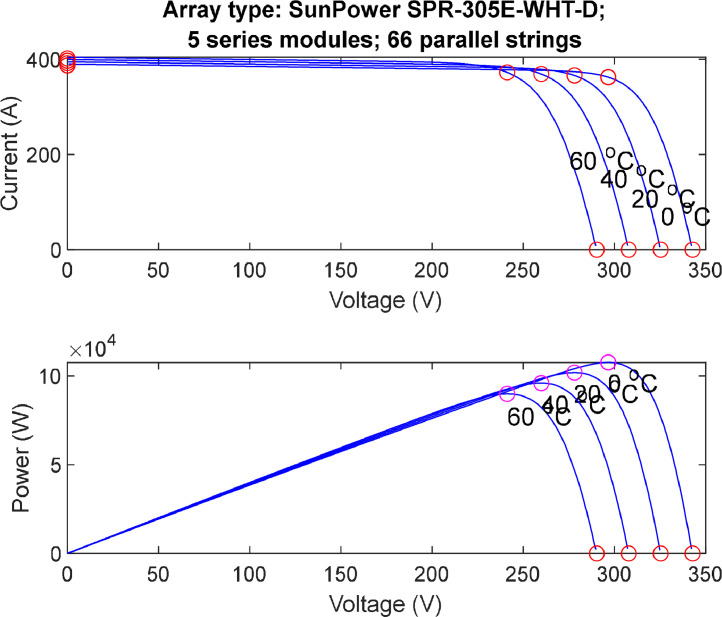
I-V & P-V characteristics (varying temperature at solar radiation ($1000\;\;W/m^{2}$)).

Fig. 6 demonstrate the effect of changing the solar arrays temperature values while have a constant 1000 W/m^2^ solar radiation applied on the PV system. Varying the temperature significantly influences the electrical characteristics of photovoltaic (PV) cells, as depicted in the I-V (current-voltage) and P-V (power-voltage) characteristics. With an increase in temperature, the electron concentration within the semiconductor material of the PV cell rises, leading to an overall boost in generated current. Additionally, the temperature affects the saturation current, a key factor in the diode equation, contributing to changes in the I-V curve. In terms of the P-V characteristics, temperature fluctuations can shift the location of the Maximum Power Point (MPP), where the power output is maximized. Higher temperatures may increase current but can simultaneously decrease voltage, influencing the efficiency of the PV cell. Manufacturers often provide a temperature coefficient to help quantify these effects, offering insights into how efficiency and output change with temperature variations.

• Partial Shading Assessment Profiles

**Fig. 7:**
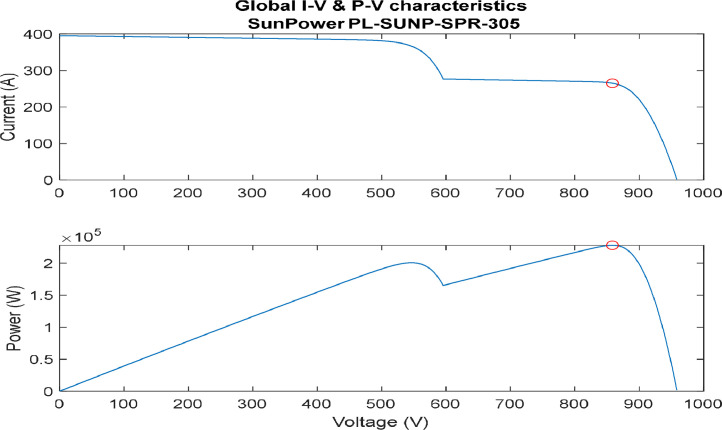
I-V & P-V characteristics (simple partial shading). (Array1 = 30%, Array2 = 0%, Array3 = 0%shades).

**Fig. 8:**
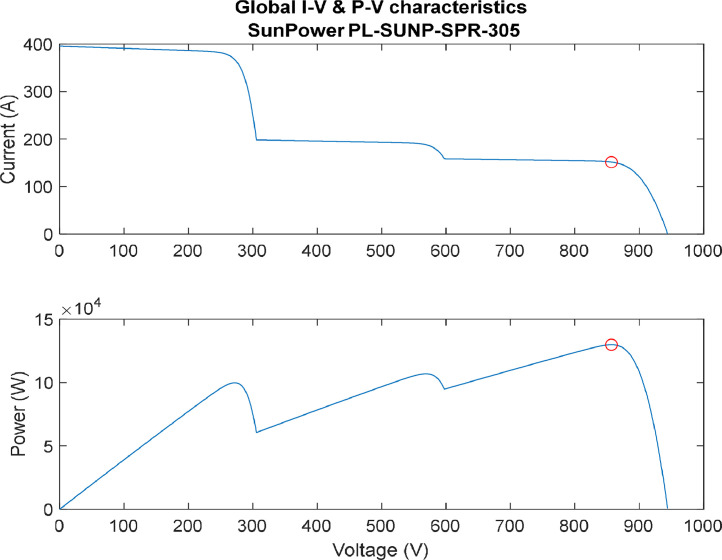
I-V & P-V characteristics (moderate partial shading). (Array1 = 0%, Array2 = 50%, Array3 = 60%shades).

**Fig. 9:**
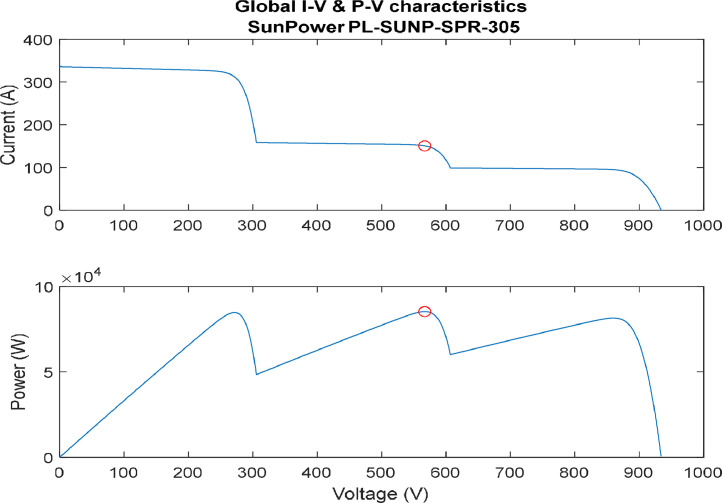
I-V & P-V characteristics (extreme partial shading). (Array1 = 15%, Array2 = 60%, Array3 = 25% shades).

**Fig. 10:**
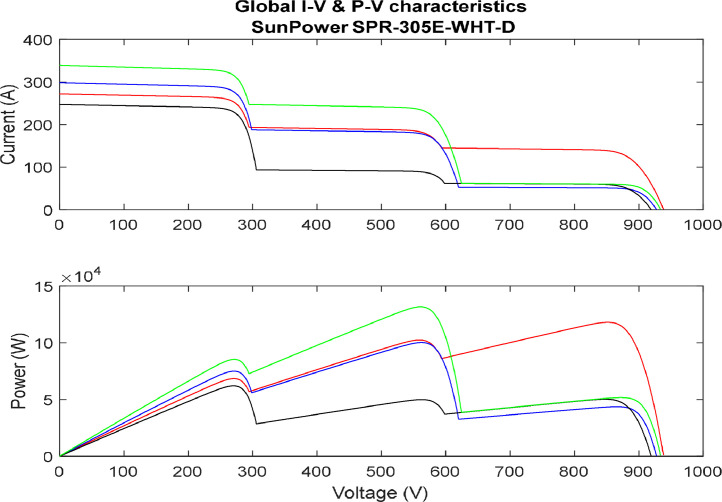
I-V & P-V characteristics (dynamic partial shading). (Partial shading is random).

Fig. 7, Fig. 8, Fig. 9, Fig. 10 provide several profiles scenarios considered in the assessment for different Partial Shading profiles. To clarify, partial shading has a pronounced effect on the performance of photovoltaic (PV) systems, with the severity and dynamics of shading profiles influencing energy output and system efficiency. In simple partial shading, localized shading of a small section results in reduced power generation, impacting overall efficiency. Moderate partial shading involves intermittent shading over larger areas, causing fluctuations in power output and affecting system stability. Severe partial shading, where extensive portions of the PV array are shaded simultaneously, leads to substantial reductions in power output, potential hotspots, and decreased reliability. Dynamic partial shading involves rapid and unpredictable changes in shading conditions over time, posing challenges for traditional Maximum Power Point Tracking (MPPT) algorithms and resulting in suboptimal energy capture. Advanced MPPT algorithms, like the Sequential Monte Carlo (SMC) algorithm, aim to mitigate these effects by optimizing energy capture in dynamic and challenging shading scenarios, ensuring efficient PV system operation.

1. Simple Partial Shading Effect.

Fig. 7, represent the I-V and P-V characteristics of a simple case partial shading profile. In this profile, two sets of the arrays have no partial shading, while one set has 30 % partial shading affecting it. As seen from the figure, two maximum points are generated while only one of them is considered a global.

2. Moderate Partial Shading Effect.

In addition, Fig. 8 illustrate the I-V and P-V characteristics of a moderate partial shading profile. In this profile, two sets of the arrays have partial shading, while one set has no partial shading affecting it. As seen from the figure, three maximum points are generated while only one of them is considered a global. Here, classical technique of locating the global maximum power point (GMPP) will have difficulties due to the ability of distinguishing the GMPP.

3. Extreme partial shading effect.

The extreme case of partial shading is represented in Fig. 9. In this profile, all arrays are effected with different partial shading values creating three maximum points. However from the figure, it is clearly seen that all maximum points are close to each other in value creating a severity in locating the GMPP. This is an important case to consider in the evaluation because it really shows the capability of the considered technique to locating the GMPP.

4. Dynamic Partial Shading Effect.

Fig. 10 represent dynamic partial shading effect. The study of Dynamic Partial Shading (DPS) effects is of paramount importance in the field of photovoltaic (PV) systems, as it addresses the challenges posed by rapidly changing and unpredictable shading conditions. DPS occurs when shadows move across a solar array due to factors like passing clouds, foliage, or nearby structures. Understanding the impact of DPS is crucial for enhancing the efficiency and performance of PV systems, as traditional Maximum Power Point Tracking (MPPT) algorithms may struggle to adapt to these dynamic scenarios. Investigating DPS effects provides valuable insights into how shading fluctuations influence the energy output, stability, and reliability of PV systems. Moreover, research on DPS contributes to the development of advanced MPPT algorithms, such as the Sequential Monte Carlo (SMC) algorithm, designed to optimize energy capture in the presence of rapid and unpredictable shading changes. This knowledge is essential for designing robust and adaptive PV systems capable of effectively harnessing solar energy in real-world, dynamic environmental conditions. It is worth noting that each color represent a different set of partial shading conditions dynamically affecting the PV system.

## 3 Experimental Design, Materials and Methods

The research study titled “Dynamic and Adaptive Maximum Power Point Tracking Using Sequential Monte Carlo Algorithm for Photovoltaic System” employed an extensive simulation-based approach to investigate the effectiveness of the proposed Sequential Monte Carlo (SMC) algorithm for enhancing maximum power point tracking (MPPT) in photovoltaic (PV) systems. The simulation process began by creating a comprehensive PV system model using Matlab/Simulink 2018 version. This model accurately captured the behaviour of the PV panels, considering factors like variations in solar irradiance, changes in ambient temperature, and the electrical characteristics of the PV panels.

Essential to the simulation design was the seamless integration of the Sequential Monte Carlo (SMC) algorithm [1]. This adaptive algorithm was incorporated into the PV system model to facilitate dynamic MPPT. This was achieved by consistently fine-tuning the operational parameters of the PV panels in response to real-time environmental data. A critical element of the simulation was the creation of realistic environmental inputs. Simulated solar irradiance, temperature, and partial shading profiles were formulated to closely mirror actual outdoor circumstances. These profiles played a vital role as essential references for assessing the authenticity of the Solar PV system. The methodology of the algorithm aimed to establish a dynamic and adaptive MPPT approach for PV systems. The SMC algorithm utilized a probabilistic framework to iteratively estimate the maximum power point of the PV panels. The adaptability and robustness of the SMC algorithm were crucial attributes that enabled it to effectively handle uncertainties in real-world scenarios. The dynamic nature of the SMC algorithm allowed it to swiftly adapt to changing solar irradiance, temperature, and partial shading conditions. This adaptability ensured continuous estimation of the optimal operating point for PV panels, thereby enabling maximum power extraction under diverse environmental circumstances.

The model employed the generated profiles of solar irradiance, temperature, and partial shading as inputs to dynamically regulate the operating point of the PV panels. This comprehensive execution aimed to assess the algorithm's performance under varying conditions. To gauge its effectiveness, multiple performance metrics were employed, including tracking speed, accuracy, efficiency, and adaptability. Comparative analyses were conducted against conventional MPPT techniques to underscore the advantages of the SMC algorithm. The outputs of the simulation, algorithm behaviour, and performance metrics underwent meticulous data analysis using Matlab/Simulink designed code and model Ref. [5]. This analysis provided valuable insights into the algorithm's dynamic response, accuracy, and efficiency across a spectrum of scenarios. The simulation-centered research design facilitated controlled experimentation and thorough evaluation of the proposed SMC algorithm's effectiveness in dynamic and adaptive MPPT for PV systems. Through the integration of the algorithm into a simulated PV system model and exposure to varying environmental inputs, this methodology offered significant insights into the algorithm's potential to optimize power capture efficiency.

• Important Matlab/Simulink Script Files

Important Matlab/Simulink function files are provided in Ref. [6].

## Limitations

None.

## Ethics Statement

The current work meets the ethical requirements for publication in Data in Brief and does not involve human subjects, animal experiments, or any data collected from social media platforms.

## CRediT authorship contribution statement

**Alhaj-Saleh A. Odat:** Conceptualization, Methodology, Software, Data curation, Visualization, Investigation, Software, Validation, Writing – original draft. **Moayyad Shawaqfah:** Investigation, Software, Validation, Data curation. **Fares Al-Momani:** Supervision, Visualization, Writing – original draft. **Bashar Shboul:** Writing – review & editing, Methodology, Validation, Writing – original draft.

## Data Availability

SMC-MPPT (Original data) (Mendeley Data).
